# Incidence and Predictors of Acute Ischemic Lesions on Brain Magnetic Resonance Imaging in Patients With a Clinical Diagnosis of Transient Ischemic Attack in China

**DOI:** 10.3389/fneur.2019.00764

**Published:** 2019-07-16

**Authors:** Junliang Yuan, Zejin Jia, Yangguang Song, Shuna Yang, Yue Li, Lei Yang, Wei Qin, Wenli Hu

**Affiliations:** ^1^Department of Neurology, Beijing Chaoyang Hospital, Capital Medical University, Beijing, China; ^2^Department of Pathology, Beijing Chaoyang Hospital, Capital Medical University, Beijing, China

**Keywords:** transient ischemic attack, ischemic stroke, diffusion-weighted imaging, ABCD3-I score, dawson score

## Abstract

**Background:** The associations between the clinical characteristics and diffusion-weighted imaging (DWI) positivity in patients with a clinical diagnosis of transient ischemic attack (TIA) are still poorly understood. The purpose of our study was to determine the incidence of TIA related acute infarction by DWI, and to determine the underlying predictors of DWI positivity in TIA patients.

**Methods:** Between Jan 2017 and Dec 2018, we retrospectively enrolled 430 patients with a clinical diagnosis of TIA who underwent DWI. Patients were divided into those with acute ischemic lesions (DWI positive group) and those without (DWI negative group). The clinical characteristics, laboratory data, and imaging parameters were compared between the two groups.

**Results:** A total of 430 time-based TIA patients (mean age, 61.4 ± 13.0) were enrolled in this study. About 126 (29.3%) of TIA patients had a DWI positive lesion in our series. Comparing TIA patients with positive DWI to those with negative DWI, acute lesions were more likely to be more male, have higher hyperlipidemia and a smoking history, more speech abnormalities and increased motor weakness; and higher systolic and diastolic blood pressure, homocysteine, fasting blood glucose, and the scores of ABCD2, ABCD3, ABCD3-I, and Dawson. Several independent predictors of DWI positivity were identified with logistic regression analysis: motor weakness (odds ratio 4.861, *P* = 0.021), speech abnormalities (odds ratio 4.029, *P* = 0.024), and ABCD3-I (odds ratio 13.141, *P* = 0.001). ABCD3-I showed the greatest area under the ROC curve, with a sensitivity of 85.7% and specificity of 72.4%.

**Conclusion:** In patients with a clinical diagnosis of TIA, 29.3% demonstrated acute DWI lesions on brain magnetic resonance imaging (MRI). They were associated with motor weakness, speech abnormalities and higher ABCD3-I score at admission.

## Introduction

Stroke is the second leading cause of death worldwide, and the first cause of death in China. About 20% of stroke is preceded by an episode of transient ischemic attack (TIA). Patients with TIA have the highest risk of early stroke especially in the first 48 h (1, 2) and subsequent adverse events (3). The time-based definition of TIA was classically defined as the presence of focal neurological symptoms ascribable to a vascular etiology lasting <24 h. The tissue-based definition of TIA was proposed to classify patients only if the symptoms fit the clinical syndrome and if no acute ischemic lesion was identified by magnetic resonance imaging (MRI) (4). Thus, diffusion-weighted imaging (DWI) has been a mandatory tool of the diagnosis and treatment of patients with TIA (5). However, it has been reported that only 11.1% of patients with TIA could be detected by DWI (6). Several studies suggested DWI positivity in TIA was associated with clinical characteristics such as age (7), longer symptom duration (8), motor deficit, aphasia, and NIHSS score (6), and large-vessel occlusion of magnetic resonance angiography (MRA) (9). However, these associations between the clinical characteristics and DWI positivity are still incompletely understood.

In resource-limited settings, clinicians need to rely on patient history, neurological examination, and patient-specific risk factors to stratify. Thus, it is worthwhile to develop some reliable risk scores to prevent stroke in an early stage after the onset of TIA. The ABCD2 score was first developed to provide a structured way to stratify the early risk of stroke after TIA (10, 11). ABCD2-I score (12), ABCD3 (13), and ABCD3-I score (14, 15) were then also widely used to stratify the risk factors of stroke. The ABCD score system was widely used, however, up to a third of mimics were also found to have ABCD2 scores ≥ 4 (16). Furthermore, the management of ABCD3-I needs the immediate stroke specialist assessment, and urgent brain MRI and vascular imaging, which will be much more difficult with higher cost-effectiveness. The Dawson score (17), while not widely used, incorporates more specific clinical characteristics of patient presentation and is designed for sensitivity, thus we sought to investigate its utility in our population.

Thus, the aims of the present study are to determine the incidence of TIA related acute lesions by DWI and to investigate its utility of ABCD score system and Dawson score in our population, and finally to determine underlying predictors of DWI positivity in TIA patients.

## Materials and Methods

### Patients

Our study was a retrospective, observational study in the Department of Neurology, Beijing Chaoyang Hospital, Capital Medical University. We retrospectively enrolled consecutive 430 patients between Jan 2017 and Dec 2018 and they all underwent brain MRI. The mean time from symptom onset to MRI was 72 h. TIA was classically defined as a focal cerebral ischemic event with neurological symptoms lasting <24 h regardless of brain lesions detected by brain imaging, including computed tomography (CT), and MRI (4). The detection of acute ischemic lesions was based on DWI. Based on DWI, patients were divided into those with acute ischemic lesions (DWI positive group) and those without (DWI negative group), and their demographics, clinical data, and imaging variables were analyzed. Ischemic stroke subtypes were classified only limited to DWI positive group according to the criteria in the Trial of Org 10172 in Acute Stroke Treatment (TOAST) (18).

### Clinical Variables

We retrospectively obtained the following clinical data: age, gender; vascular risk factors including hypertension, diabetes mellitus, hyperlipidemia, stroke, coronary heart disease, atrial fibrillation, and current smoking. The score s of ABCD2, ABCD3, ABCD3-I, and Dawson were calculated (Tables 1, 2). The laboratory blood tests were obtained from medical records, including the counts of red blood cell, white blood cell, platelet; hemoglobin, fibrinogen, D-dimer, fasting blood glucose (FBG), hemoglobin A1c, glycated albumin, uric acid, homocysteine, total cholesterol, low-density lipoprotein, high-density lipoprotein, and C-reactive protein. The presenting symptoms of TIA included unilateral motor weakness, sensory disturbance, speech abnormalities (dysarthria/aphasia), dizziness, loss of consciousness, hemianopia, and amnesia. Duration of TIA was classified into 3 categories: <10, 10–59 min, or more than 1 h.

**Table 1:** ABCD2, ABCD3, and ABCD3-I scores.

**Variable**	**ABCD2**	**ABCD3**	**ABCD3-I**
Age ≥60 years	1	1	1
Blood pressure ≥140/90 mm Hg	1	1	1
**Clinical features**			
Speech impairment without weakness	1	1	1
Unilateral weakness	2	2	2
**Duration, min**			
10–59	1	1	1
≥60	2	2	2
Diabetes mellitus	1	1	1
Prior TIA within 1 week	NA	2	2
**Imaging**			
Stenosis on carotid imaging (≥50%)	NA	NA	2
Abnormal DWI	NA	NA	2
Total	0–7	0–9	0–13

*NA, not applicable*.

**Table 2:** TIA scoring system.

**Variable**	**Score if yes**	**Score if no**	**Std error (*P*-value)**
History of stroke or TIA	0.5	0	0.1 (3.5 × 10^−7^)
Headache	0	0.5	0.11 (7.1 × 10^−5^)
Diplopia	1.2	0	0.28 (2.7 × 10^−6^)
Loss of consciousness/Pre-syncope	0	1.1	0.21 (1.9 × 10^−7^)
Seizure	0	1.6	0.43 (1.4 × 10^−4^)
Speech abnormalities	1.3	0	0.14 (<1 10^−10^)
Unilateral limb weakness	1.7	0	0.10 (<1 × 10^−10^)
Upper motor neuron facial weakness	0.6	0	0.15 (9.5 × 10^−8^)
Age	Multiply by 0.04		0.004 (<1 × 10^−10^)

*All values reflect the regression coefficients seen and P-values refer to the significance of each variable in the final model. Std Error, Standard Error. To calculate the score, all values should be summed. If total score >6.1, classify as TIA. For “2:1 cost ratio,” if total score >5.4, classify as TIA*.

### Imaging Data

The sequences of MRI included T1-weighted images, T2-weighted images, fluid-attenuated inversion recovery images, DWI, and the accompanying MRA with 3-dimensional time-of-flight images were obtained. The presence of acute ischemic lesions was defined by areas of high signal intensity on DWI. All neuroimaging data and clinical reports were reviewed blindly by neurologists and neuroradiologists, without prior knowledge of baseline clinical data. For analysis of MRA, intracranial as well as extracranial stenoses >50% were considered to be significant and if the stenosis was in a territory appropriate to the patient's symptom, it was defined as the “MRA abnormality.”

### Treatment

We retrospectively examined the content of antithrombotic treatments administered after admission, including anticoagulant treatment, antiplatelet treatment (aspirin or clopidogrel), and administered either as a single or dual treatment.

### Statistical Analysis

The data were described using mean and standard deviation, or median and interquartile range values for continuous variables, and absolute numbers and percentages for nominal and categorical variables, and we compared the groups using the non-parametric *Mann-Whitney U*-test. We performed a *chi-square* test to determine the correlation between categorical variables and a *t*-test between continuous variables. The multivariate logistic regression analysis was performed to find predictors for the presence of DWI lesions. The receiver operating characteristic curve (ROC) analysis was performed to discriminate DWI-positive from DWI-negative patients using the value of area under the curve (AUC). We used the Statistical Package for Social Sciences (SPSS) version 16.0 (SPSS Inc., Chicago, IL, USA) for data analysis. A *P* value <0.05 was considered statistically significant.

## Results

Among a total of 430 patients with time-defined TIA, the mean age was 61.4 ± 13.0 years, and 69.5% were male. One hundred twenty-six (29.3%) patients had high-intensity lesions on DWI. We classified the patients with positive DWI as stroke etiology according to the TOAST: 48 (38.1%) of large-artery atherosclerosis, 14 (11.1%) of cardioembolism, 40 (31.7%) of small artery occlusion, 4 (3.2%) of other determined etiology, 20 (15.9%) of undetermined etiology.

Table 3 showed the demographics, baseline clinical characteristics, vascular risk factors, TIA symptoms, treatment of all TIA patients (*N* = 430), DWI positive group (*N* = 126), and DWI negative group (*N* = 304). There were significant differences in sex, systolic and diastolic blood pressure, hypercholesterolemia and smoking history, motor weakness, speech abnormalities, acute antithrombotic treatment, and the scores of ABCD2, ABCD3, ABCD3-I, and Dawson between the two groups (*P* < 0.05). The percentage of patients who were medicated with dual antiplatelet therapy was significantly higher in the DWI positive group than DWI negative group (61.9 vs. 10.2%, *P* < 0.001). There were no significant differences in the other baseline clinical characteristics and MRA abnormality between the two groups.

**Table 3:** The baseline demographics, clinical characteristics in TIA patients.

**Variables**	**Total TIA (*N* = 430)**	**DWI positive (*N* = 126)**	**DWI negative (*N* = 304)**	***P***
**Demographics**				
Age (Y)	61.4 ± 13.0	61.1 ± 13.4	61.5 ± 12.8	0.789
Sex (Male, %)	306 (69.5%)	100 (79.4%)	206 (67.8%)	0.016^*^
Systolic BP	146.3 ± 20.7	151.7 ± 18.3	144.2 ± 21.4	0.001^*^
Diastolic BP	83.4 ± 13.2	87.5 ± 12.7	81.7 ± 13.2	0.001^*^
**Risk factors**				
Hypertension	227 (63%)	79 (62.7%)	198 (65.1%)	0.631
Diabetes mellitus	130 (29.5%)	40 (31.7%)	90 (29.6%)	0.660
Coronary disease	46 (10.6%)	9 (7.1%)	37 (12.2%)	0.125
Prior stroke	82 (18.6%)	29 (23%)	53 (17.4%)	0.180
Hypercholesterolemia	306 (69.5%)	99 (78.6%)	207 (68.1%)	0.029^*^
Atrial fibrillation	15 (3.4%)	4 (3.2%)	11 (3.6%)	0.819
Smoking	216 (49.1%)	81 (64.3%)	135 (44.4%)	0.001^*^
**Clinical features**				
Motor weakness	254 (57.7%)	97 (77%)	157 (51.6%)	0.001^*^
Sensory disturbance	126 (28.6%)	39 (31%)	87 (28.6%)	0.628
Speech abnormalities	162 (36.8%)	73 (57.9%)	89 (29.3%)	0.001^*^
Diplopia	21 (4.8%)	6 (4.8%)	15 (4.9%)	0.940
Dizziness	140 (31.8%)	81 (35.7%)	209 (31.2%)	0.369
Loss of consciousness	36 (8.2%)	5 (4%)	31 (10.2%)	0.034^*^
Hemianopia	14 (3.2%)	4 (3.2%)	10 (3.3%)	0.951
Amnesia	10 (2.3%)	2 (1.6%)	8 (2.6%)	0.513
**Duration**				0.646
<10 min	182 (41.4%)	50 (39.4%)	132 (44.6%)	
10–59 min	168 (38.2%)	53 (42.1%)	115 (38.9%)	
1 h	72 (16.4%)	23 (18.3%)	49 (16.6%)	
**Treatment**				0.001^*^
Aspirin	164 (37.3%)	33 (26.2%)	131 (43.1%)	
Clopidogrel	84 (19.1%)	13 (10.3%)	71 (23.3%)	
Dual antiplatelet therapy	109 (24.8%)	78 (61.9%)	31 (10.2%)	
Anticoagulation	8 (1.8%)	2 (1.6%)	6 (2%)	
*MRA abnormality*	26 (6.0%)	6 (4.8%)	20 (6.6%)	0.472
**TIA scores**				
ABCD2	4 (3 ~ 5)	4 (3 ~ 5)	3 (2.25 ~ 4)	0.001^*^
ABCD3	5 (3 ~ 6)	6 (4 ~ 7)	4 (3 ~ 5)	0.001^*^
ABCD3-I	5 (4 ~ 7)	8 (6 ~ 9)	5 (3 ~ 6)	0.001^*^
Dawson	7.2 (6.1 ~ 8.1)	7.68 (6.97 ~ 8.72)	6.83 (5.76 ~ 7.93)	0.001^*^

*Data were presented as median with interquartile range in parentheses or number with the percentage in parentheses*.

* *P <0.05*.

*TIA, transient ischemic attack; DWI, diffusion-weighted imaging; BP, blood pressure*.

The laboratory findings were presented in Table 4. The levels of homocysteine and FBG were significantly higher in DWI-positive group than those in DWI-negative group. There were no differences in the other laboratory data between the two groups.

**Table 4:** The laboratory findings and the risk scores of TIA in TIA patients.

**Variables**	**Total TIA (*N* = 430)**	**DWI positive (*N* = 126)**	**DWI negative (*N* = 304)**	***P***
WBC (^*^10^9^/L)	7.1 ± 2.3	7.1 ± 2.3	7.0 ± 2.3	0.752
RBC (^*^10^12^/L)	4.5 ± 0.6	4.5 ± 0.7	4.5 ± 0.5	0.623
HGB (g/L)	141.8 ± 17.0	1435 ± 15.8	141.0 ± 17.4	0.289
PLT (^*^10^9^/L)	212.4 ± 57.9	195.4 ± 48.6	219.6 ± 60.0	0.248
CHOL (mmol/L)	4.5 ± 1.0	4.4 ± 0.9	4.6 ± 1.14	0.119
LDL (mmol/L)	2.7 ± 0.9	2.6 ± 0.8	2.7 ± 0.9	0.248
HDL (mmol/L)	1.1 ± 0.4	1.1 ± 0.3	1.2 ± 0.4	0.265
Uric acid (μmol/L)	336.2 ± 86.6	331.9 ± 91.2	338.1 ± 84.7	0.502
Homocysteine (μmol/L)	17.7 ± 10.5	20.1 ± 11.5	16.8 ± 10	0.01^*^
FBG (mmol/L)	6.6 ± 3.1	7.3 ± 3.4	6.4 ± 30	0.01^*^
HbA1c (%)	6.3 ± 1.3	6.4 ± 1.5	6.3 ± 1.3	0.413
Glycated albumin (%)	15.5 ± 4.3	14.3 ± 2.7	16.4 ± 5.1	0.201
Fibrinogen (mg/dl)	271.8 ± 76.9	266.3 ± 78.3	274.2 ± 76.4	0.591
CRP (mg/L)	1.5 (0.8 ~ 3)	2 (1.1 ~ 3.1)	1.5 (1.1 ~ 2.3)	0.070

*Values represent the number (percent) for categorical variables and mean ± SD or median (interquartile range) for continuous variables*.

* *P <0.05*.

*TIA, transient ischemic attack; DWI, diffusion-weighted imaging; RBC, red blood cell; WBC, white blood cell; PLT, platelet; HGB, hemoglobin; FBG, fasting blood glucose; HbA1c, hemoglobin A1c; CHOL, cholesterol; LDL, low-density lipoprotein; HDL, high-density lipoprotein; CRP, C-reactive protein*.

Logistic regression analysis was shown in Table 5, and we found motor weakness (odds ratio 4.861, 95% *CI* 1.264–18.344, *P* = 0.021), speech abnormalities (odds ratio 4.029, 95% *CI* 1.203–13.496, *P* = 0.024) and ABCD3I (odds ratio 13.141, 95% *CI* 7.392–23.360, *P* = 0.001) were independently associated with positive DWI lesions.

**Table 5:** Associated factors with acute infarction detected by DWI using logistic regression analysis.

**Predictors**	***B***	***P***	***OR***	**95%** ***CI*** **for** ***OR***	
				**Lower**	**Upper**
Motor weakness	1.572	0.021^*^	4.816	1.264	18.344
Speech abnormalities	1.393	0.024^*^	4.029	1.203	13.496
ABCD3-I	2.576	0.001^*^	13.141	7.392	23.360

* *P <0.05*.

*CI, confidence interval; OR, odds ratio; DWI, diffusion-weighted imaging*.

**Figure 1:**
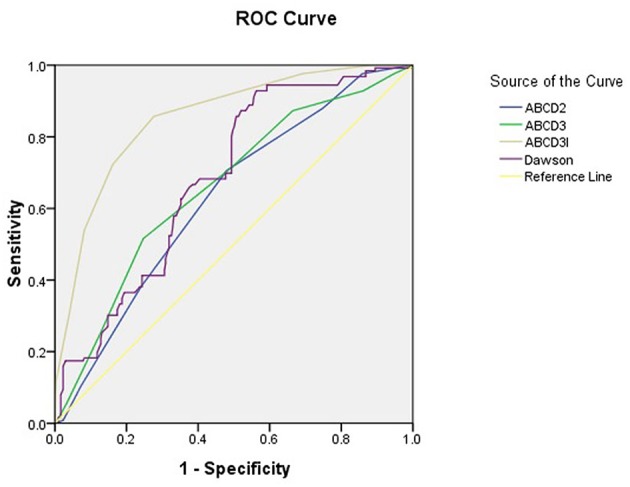
The ROC of different scales for the prediction of DWI positivity in TIA patients.

The area under the ROC curve (95% *CI*) of the scores of ABCD2, ABCD3, ABCD3-I, and Dawson for the prediction of acute ischemic events were 0.629 (95% *CI*, 0.573–0.684), 0.660 (95% *CI*, 0.604–0.716), 0.854 (95% *CI*, 0.815–0.892), 0.684 (95% *CI*, 0.633–0.736), respectively. Figure 1 showed the ROC for selected predictors of DWI positive TIA patients. ABCD3-I showed the greatest area under the ROC curve, with the sensitivity of 85.7% and specificity of 72.4%, respectively.

## Discussion

In our present study, we found 29.3% of TIA patients had a positive DWI lesion on brain MRI in patients with clinically diagnosed TIA. They were independently associated with motor weakness, speech abnormalities, and higher ABCD3-I score at admission. ABCD3-I showed the greatest area under the ROC curve (0.854), and Dawson was 0.684.

DWI has been a mandatory tool in the diagnosis of a TIA or acute ischemic stroke (19). DWI is more sensitive than CT for detecting acute ischemia, and up to one-third of patients diagnosed with TIA were found to have an acute infarct on DWI (20). The frequency of positive DWI findings varied from 9 to 67% among different cohorts of TIA (12, 21, 22). A meta-analysis of the 9,078 patients in 47 studies showed that 34.3% of patients had a DWI lesion (23). The incidence of DWI positivity in our series was similar to previous reports (9, 23, 24). It has been reported that one of the important factors that affect DWI positivity rate was the duration from stroke onset to MRI examination (25). In our present study, the mean time from symptom onset to MRI was 72 h. It is probable that if patients receiving MRI within the first 24–48 h were more likely to have acute DWI lesions than patients receiving MRI after 72 h.

In resource-limited settings, the clinical evaluation could be quite important to stratify different risk factors of TIA patients. In patients with TIA, symptom duration of more than 1 h, motor deficits, and aphasia were independently correlated with detecting an abnormality with DWI (21). Subcortical acute lesions were also reported in patients with TIA, that were associated with recurrent episodes, dysarthria, and motor weakness (26). The data from Germany also indicated that the evidence of acute infarction by DWI in TIA patients was detected in 11.1 % of patients and associated with motor weakness, aphasia, and NIHSS score of ≥10 at admission (6). Our results were also consistent with these previous findings (6, 21, 24, 26, 27), especially positive DWI in TIA patients performed with the motor weakness and speech abnormalities (27). We also found that ABCD3-I showed the greatest area under the ROC curve. It was reported that the area under the ROC curve of ABCD2 score and ABCD3-I score was 0.50 and 0.58 in South Korea population (28). Our study was also in line with this study (15, 29). As for the Dawson score, we found that the AUC for the Dawson score was 0.684 in our study, which was also quite similar to the population from the United Kingdom (30). However, before applying the ABCD system or Dawson score as risk stratification, the physician should always keep in mind to never neglect the individual examination of TIA patient (31).

Our study had several limitations. First, this study was designed as a retrospective study from a single center. A study with a larger number of patients from multiple centers in China is urgently needed to confirm our results. Second, it has been reported that patients with acute DWI lesions more often performed a cardioembolic etiology or large artery atherosclerosis cause (32). As for the etiology of the TIA, however, we only classified the TIA patients with positive DWI group according to the TOAST, which were not performed in all the TIA patients. Third, we did not assess the risk factors of the recurrence of TIA or stroke, lack of subgroup analysis such as low, medium, and high risk according to ABCD2/ABCD3/ABCD3-I scores. Fourth, another limitation was the lack of data regarding prior treatment and follow-up in our study. However, the strengths of the study were listed as follows. First, as far as we know, the present study is the largest ever reported MRI including DWI investigation of TIA that identifies clinical predictors for evidence of acute infarction in patients suffering from TIA in China. Secondly, we found the levels of homocysteine and FBG were significantly higher in the DWI-positive group compared to DWI-negative group, which was a novelty in this study. Third, we further validated its utility of Dawson score in a Chinese population.

## Conclusion

In summary, our findings demonstrated that an acute infarction was detected in 29.3% of clinical diagnosed TIA patients, and it was independently associated with motor weakness, speech abnormalities, and higher ABCD3-I score at admission. A larger number of patients from multiple centers in China are urgently needed to confirm our findings, especially higher ABCD3-I score as predictors of positive DWI.

## Data Availability

The datasets analyzed in this manuscript are not publicly available. Requests to access the datasets should be directed to wenlihu3366@126.com.

## Ethics Statement

The studies involving human participants were reviewed and approved by Ethics Committee of Beijing Chaoyang Hospital, Capital Medical University. The patients/participants provided their written informed consent to participate in this study. Written informed consent was obtained from the individual(s) for the publication of any potentially identifiable images or data included in this article.

## Author Contributions

JY and WH conceived and designed the experiments. JY analyzed the data and drafted the manuscript. ZJ, YS, SY, YL, LY, and WQ collected data. All authors have read and approved the final manuscript to be published.

### Conflict of Interest Statement

The authors declare that the research was conducted in the absence of any commercial or financial relationships that could be construed as a potential conflict of interest.
